# Experimental and Numerical Analysis of Refill Friction Stir Spot Welding of Thin AA7075-T6 Sheets

**DOI:** 10.3390/ma14237485

**Published:** 2021-12-06

**Authors:** Venkata Somi Reddy Janga, Mokhtar Awang, Mohd Fadillah Yamin, Uceu F. H. Suhuddin, Benjamin Klusemann, Jorge F. dos Santos

**Affiliations:** 1 Department of Mechanical Engineering, Universiti Teknologi PETRONAS, Seri Iskandar 32610, Perak, Malaysia; venkata_19001587@utp.edu.my (V.S.R.J.); mohdfadillakyamin@gmail.com (M.F.Y.); 2 Solid State Materials Processing, Institute of Materials Mechanics, Helmholtz-Zentrum Hereon, Max-Planck-Str. 1, 21502 Geesthacht, Germany; uceu.suhuddin@hereon.de (U.F.H.S.); benjamin.klusemann@hereon.de (B.K.); jorge.dos.santos@hereon.de (J.F.d.S.); 3 Institute of Product and Process Innovation, Leuphana University of Lüneburg, Universitätsallee 1, 21335 Lüneburg, Germany

**Keywords:** refill friction stir spot welding, temperature measurements, microstructure analysis, hardness measurement, simulation of joining process, friction stir spot welding, DEFORM-3D, finite element modelling

## Abstract

The refill friction stir spot welding (refill FSSW) process is a solid-state joining process to produce welds without a keyhole in spot joint configuration. This study presents a thermo-mechanical model of refill FSSW, validated on experimental thermal cycles for thin aluminium sheets of AA7075-T6. The temperatures in the weld centre and outside the welding zone at selected points were recorded using K-type thermocouples for more accurate validation of the thermo-mechanical model. A thermo-mechanical three-dimensional refill FSSW model was built using DEFORM-3D. The temperature results from the refill FSSW numerical model are in good agreement with the experimental results. Three-dimensional material flow during plunging and refilling stages is analysed in detail and compared to experimental microstructure and hardness results. The simulation results obtained from the refill FSSW model correspond well with the experimental results. The developed 3D numerical model is able to predict the thermal cycles, material flow, strain, and strain rates which are key factors for the identification and characterization of zones as well for determining joint quality.

## 1. Introduction

Friction stir spot welding (FSSW) [1] is a solid-state joining technology largely used for aluminium alloys. FSSW is a potential alternative to conventional joining processes like riveting and resistant spot welding. As solid-state joining processes operate below the melting point of the materials, problems like liquation cracking and porosity that arise in conventional fusion welding are avoided [2]. The most well-known solid-state joining process is probably friction stir welding (FSW). FSW involves tool plunging which is a perpendicular motion of the tool into the workpiece and transverse along the path of the weld. Similar to FSW, FSSW also utilizes the heat originated by friction between the workpiece and the tool, however, unlike FSW, FSSW is restricted to plunging and retracting the tool along a vertical path. FSSW leaves out an exit hole as part of the material is squeezed out of the weld spot. The FSSW approaches utilized currently can be characterized in four fundamental categories: FSSW [1], refill FSSW [3], swept FSSW [4], and swing FSSW [5]. For an overview of the different FSSW variants, the interested reader is referred to Padhy et al. [6].

**Figure 1:**
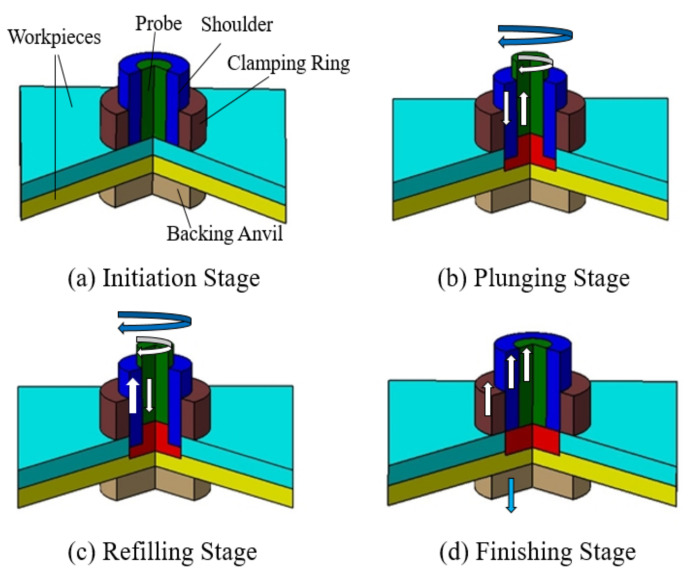
Refill friction stir spot welding (FSSW) process: (**a**) initiation stage—clamping of workpieces; (**b**) shoulder-plunging stage; (**c**) refilling stage; (**d**) finishing stage.

Refill FSSW, resulting in spot weld without exit hole, was developed and patented by Helmholtz Zentrum Hereon [3]. Refill FSSW tool setup comprises a shoulder, probe, and clamping ring with an additional backing anvil. The shoulder and probe can move independently in terms of rotational and translational movement. The refill FSSW process can be categorized according to the movement sequences of the rotating tool parts. In the shoulder plunging variant, shown in Figure 1, the shoulder plunges into the workpiece, wherein the probe plunging variant the probe plunges [7]. The shoulder plunging variant is the often preferred process, as it produces a larger welding area [8,9,10,11,12,13]. Another recent variant is the differential rotation refill FSSW [14], where the rotational speed and direction between both tool parts can be different.

The refill FSSW process stages according to the shoulder plunging variant are as follows:

1. In the initiation stage, the probe and shoulder rotate above the workpieces, which are tightly clamped.
2. The shoulder plunges into the workpieces and the probe moves in the direction opposite to the shoulder creating a room (reservoir) for the displaced material. The rotating tool introduces severe plastic deformation and frictionally heat the material.
3. The movement of the shoulder and probe is reversed after the specified plunge depth is reached. The probe drives the displaced material back into the reservoir of the workpiece.
4. The probe and shoulder reach their initial positions leaving the weld without an exit hole. The tools are removed and the clamping force is released in the finishing stage.

The refill FSSW process involves intense deformations, frictional contact, and elevated temperatures. It is crucial to understand the physics involved in refill FSSW to optimize process parameters, designing tools, and producing high-strength welds. Most refill FSSW investigations rely on experimental studies [10,15,16,17]. However, experimental investigations during refill FSSW cannot provide a deep insight into the temperature distribution, plastic strains, and strain rates. Only a few investigations have been carried out in terms of numerical modelling of refill FSSW. Muci-Küchler et al. [18] developed a fully coupled thermo-mechanical FEM model using Abaqus-Explicit to foresee the temperature and material flow in the plunging stage of the probe plunging variant of the refill FSSW process. Ji et al. [19,20] simulated material flow in refill FSSW using Ansys-Fluent. Their CFD model focused on the material flow at different rotating speeds and plunging depths during refill FSSW. The material flow is enhanced with the increase of rotational speed and maximum flow velocities were observed near the shoulder outer periphery region. Malik et al. [21] used DEFORM-3D for refilling the exit hole in a three-stage operation using diverse types of non-consumable tools in each operation. The refilling of the exit hole was accomplished, but no quantitative results were presented. Kubit et al. [22] developed an axisymmetric thermo-mechanical model of refill FSSW. A temperature-dependent shear friction coefficient model is utilized to compensate for the incipient melting and tool slippage in the process. Zhang et al. [23] used a Coupled Eulerian-Lagrangian (CEL) formulation in ABAQUS to develop a numerical model of refill FSSW. In the study, the thermal field and material flow results of refill FSSW process of AZ91D magnesium alloy were presented and validated to experiment data, where the hook defect formation could be explained by simulation results of material flow.

Deform-3D with updated Lagrangian formulation was used for modelling of FSSW in earlier studies [24,25,26]. Although a large number of experimental research has been carried out on refill FSSW, the literature on numerical modelling of refill FSSW is still very limited. The objective of the study is to develop a valid 3D thermo-mechanical model for the refill FSSW process to predict the temperatures, material flow, including material flow velocities, plastic strain, and strain rates during the joining of thin aluminium sheets. In the current study, a fully coupled thermo-mechanical FEM refill FSSW model including plunging and refill stages is set up using an updated Lagrangian formulation available in DEFORM-3D. Experimental and numerical temperature histories are compared at specific locations, followed by a discussion of the material flow during plunging and refilling stages using point tracking in the weld zone. Experimental microstructure and hardness results are connected to the obtained simulation results.

## 2. Materials and Methods

### 2.1. Refill Friction Stir Spot Welding (FSSW) Experiment

**Figure 2:**
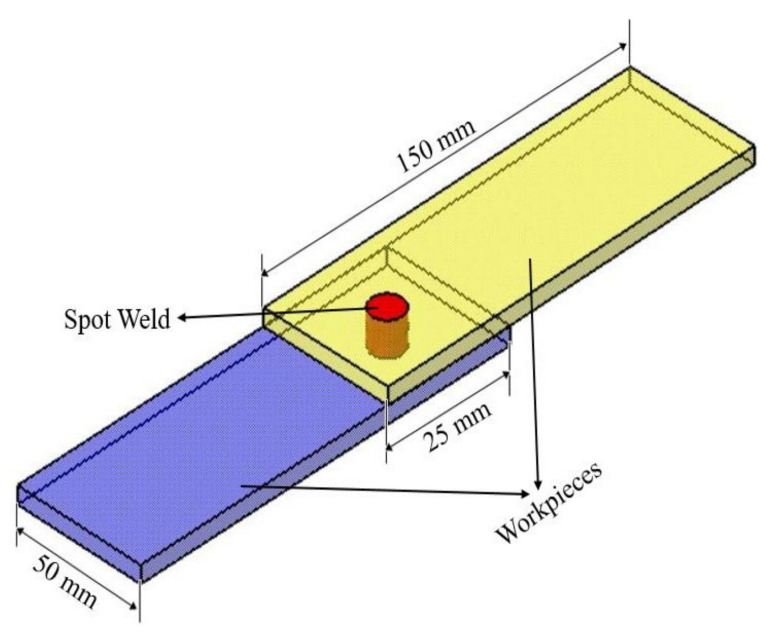
Lab shear joint configuration of similar AA7075-T6 plates.

The material for the thin sheets in this investigation is the aluminium alloy 7075-T6 with 150 mm × 50 mm × 0.6 mm. The nominal chemical composition of AA7075-T6 is 0.03Mn, 0.20Cr, 2.32Mg, 1.39Cu, 5.39Zn, 0.16Fe, Al bal. (wt%) [10]. The thin sheets are spot welded in the centre of overlapped area in a lap-shear joint configuration as shown in Figure 2.

**Figure 3:**
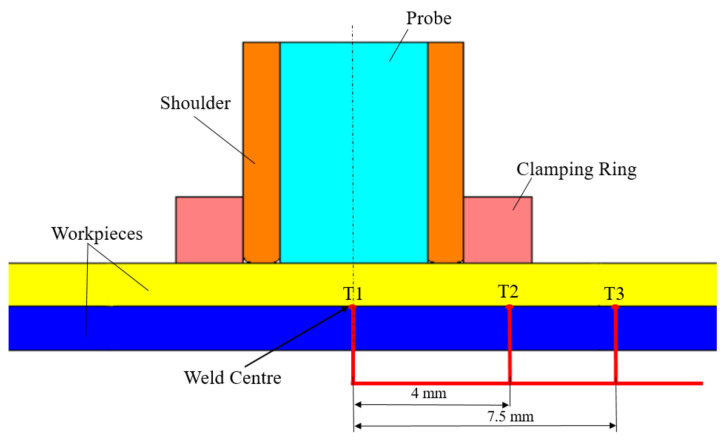
Thermocouple locations for temperature calculation during the experiment.

The refill FSSW at the spot was performed using the RPS 100 Refill FSSW machine produced by Harms and Wende GmbH, Germany. The non-consumable toolset includes a backing plate, clamping ring, shoulder, and probe. Due to high resistance for thermal fatigue cracking, tools made of hotvar material were utilized for welding. The outer diameters of the clamping ring, shoulder, and probe were 9, 6, and 4 mm, respectively. The depth of the shoulder plunge and welding times were 0.7 mm and 2.8 s, respectively, and the rotational speed was maintained stable at 3000 rpm during the process. The plunging and refilling stages are performed within 1.4 s each. K-type thermocouples (Weller, Besigheim, Germany) were utilized to record the temperature, placed between the sheets at selected locations, see T1, T2 and T3 in Figure 3. The thermocouples were installed in small holes drilled in the lower workpiece samples at marked locations. The welds were sectioned through the centre, polished, and etched to disclose the microstructure of the weld using a digital microscope- Keyence series VHX 6000 (Keyence cor., Osaka, Japan). DuraScan 80 G5 (EMCO-TEST Prüfmaschinen GmbH, Kuchl, Austria) micro-hardness tester was employed to perform Vickers hardness tests within the cross-sections. During hardness testing, a 50 g load was applied for 9 s. Hardness profiles were measured at the centre of both sheets with an interval of 0.25 mm between the test points.

### 2.2. Refill FSSW FEM Model

#### 2.2.1. Geometry

**Figure 4:**
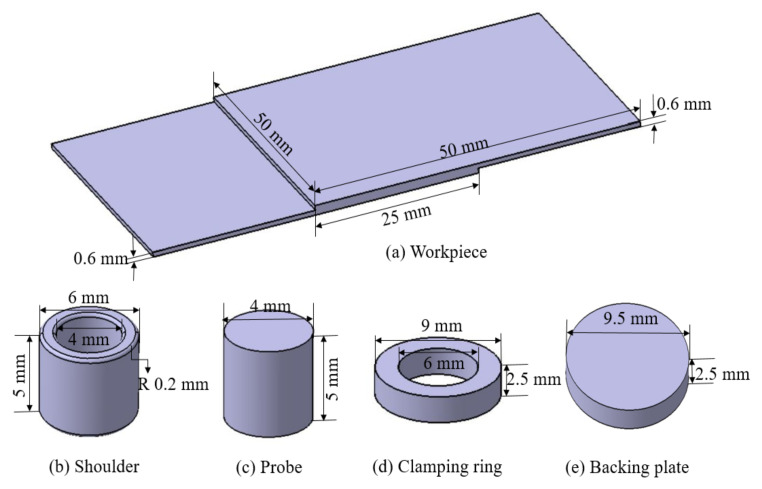
Geometries of modelled parts. (**a**) Workpiece; (**b**) shoulder; (**c**) probe; (**d**) clamping ring; (**e**) backing plate.

The geometries of the workpiece, shoulder, probe, clamping ring and backing plate were modelled using CATIA and subsequently imported to DEFORM-3D. The dimensions are given in Figure 4 for convenience. The shoulder, probe, clamping ring, and backing plate were considered as rigid bodies in the simulation. To avoid stress concentration during the shoulder plunging stage, a fillet of 0.2 mm was added to the shoulder bottom edges to increase the surface contact area, which is common practice [18]. Since the clamping ring and backing plate keep both sheets in very tight contact during the refill FSSW process, assuming no movement or friction, the two sheets are represented in the model as a single sheet. The refill welding zone is at the centre of the overlapping area. The material flow and plastic deformation are generally limited to the weld zone and a narrow area around the weld zone. In addition to that only temperatures in the weld zone is point of interest in this process as softening of material and joining due to thermal cycles happen in this region. Also, the thermal cycles outside the weld zone leads to residual stresses. Considering the above reasons and preliminary observations the length of the workpiece in this numerical model is limited. This will reduce the computational time without compromising with the accuracy of results.

#### 2.2.2. Material Model

It is essential to select an appropriate constitute law that reflects the interaction of flow stress with temperature, strain rate, and plastic strain to model the material response in refill FSSW. The Johnson–Cook model has been widely used for solid-state joining processes [27] as it accounts for strain, strain rate, and temperature effects. The Johnson–Cook flow stress model is conveyed as

(1) $$\sigma_{y}=\left[\;A+B\;{\left[\varepsilon^{pl}\right]}^{n}\;\right]\;\left[1\;+\;C\;ln\left(\frac{{\dot{\varepsilon }}^{pl}}{{\dot{\varepsilon }}^{0}}\right)\;\right]\;\left[1\;-\;{\left[\frac{T-T_{r}}{T_{m}-T_{r}}\right]}^{m}\right],$$

where $\sigma_{y}$ represents the material flow stress, $\varepsilon^{pl}$ is the equivalent plastic strain, ${\dot{\varepsilon }}^{pl}$ the plastic strain rate, ${\dot{\varepsilon }}^{0}$ the reference plastic strain rate, $T_{m}$ the melting and $T_{r}$ the ambient reference temperature. The material constants obtained at the reference strain rate are the quasi-static yield strength of the material A, strain hardening constant B, strain hardening coefficient n, and the thermal softening coefficient m. C denotes the strengthening coefficient, describing the strain-rate dependency. The Johnson–Cook coefficients reported for AA7075-T6, reported in the earlier study [28] were used in the numerical model.

Material constants for the Johnson–Cook material model for AA7075-T6 for the reference strain rate ${\dot{\varepsilon }}^{0}$ = 5 · 10^−4^/s and temperature $T_{r}=20$ °C, i.e., 293.15 K [28]. Further material properties include Young’ s modulus *E* = 68,900 MPa, Poisson’s ratio υ = 0.3, thermal expansion coefficient $\alpha =2.2\cdot 10^{-5}$/K, thermal conductivity λ= 180.175 W/m K and specific heat capacity $c_{p}=$ 870 J/kg K.

#### 2.2.3. Meshing, Contact, and Simulation Controls

**Figure 5:**
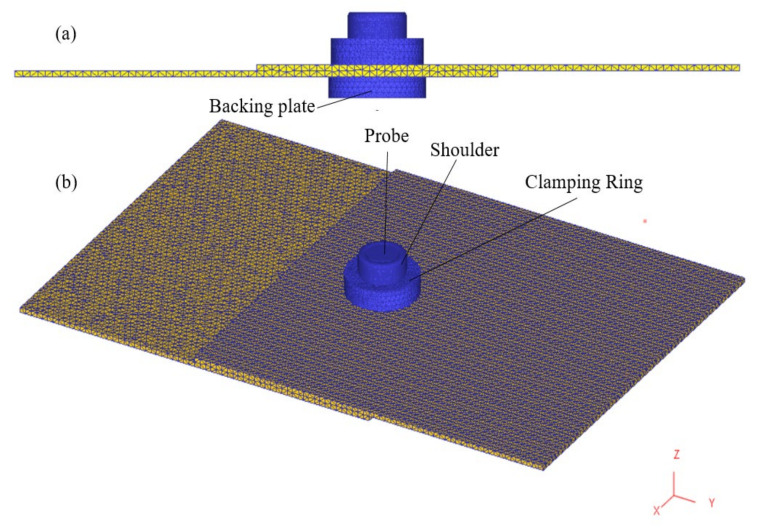
Meshed model of the refill FSSW process setup. (**a**) Side view; (**b**) isometric view.

The workpiece was meshed with approximately 180,000 elements, the shoulder with 50,000, the probe with 50,000, the clamping ring, and the backing plate with 10,000, see Figure 5. Surface meshing with a finer internal meshing scheme was used for the workpiece. Since the process involves severe plastic deformation, extensive remeshing is needed, available in DEFORM. Automatic remeshing of 0.25 mm relative interference penetration distance is assigned. Remeshing is triggered when an element edge of the workpiece is penetrated by the shoulder by the specified interference depth. However, automatic remeshing also occurred much before the specific interference depth is reached, i.e., when the elements are extremely distorted and mesh become unusable (negative Jacobian).

A temperature-dependent Coulomb friction coefficient µ was employed in the simulation, as shown in Table 1. A constant interface heat transfer coefficient of $11\;\frac{N}{mm\;s\;K}$ was applied for contact interfaces between workpiece-tools [29]. The convective heat transfer coefficient h = 0.02 $\frac{N}{mm\;s\;K}$ [30] was employed to define heat transfer between tools/workpiece and environment. The emissivity in the model is assumed as ϵ= 0.7 [31].

The two main stages of the process in the simulation are briefly explained as follows:

1. Plunging stage: the rotating shoulder plunges with a plunging speed of 0.5 mm/s and with a rotational speed of 3000 rpm until a depth of 0.7 mm, representing a total time of 1.4 s for the plunging stage. The probe, with the plunging speed of 0.625 mm/s and same rotational speed of the shoulder, moves in the opposite direction. During this stage, the deformed material from the workpiece is pushed by the shoulder into reservoir created by the upward movement of the probe.
2. Refilling stage: shoulder and probe reverse their direction with the same axial speed as in the plunging stage, ensuing the refilling of the material into the weld.

The step increment for the process was taken as 0.001 s. An updated Lagrangian incremental formulation, where the domain is updated incrementally, was used in the simulation. The point tracking scheme, available in DEFORM-3D for post-processing, was employed to track the temperatures at three characteristic points, i.e., points initially in the centre, 4 mm, and 7.5 mm away from the weld centre.

## 3. Results and Discussion

**Figure 6:**
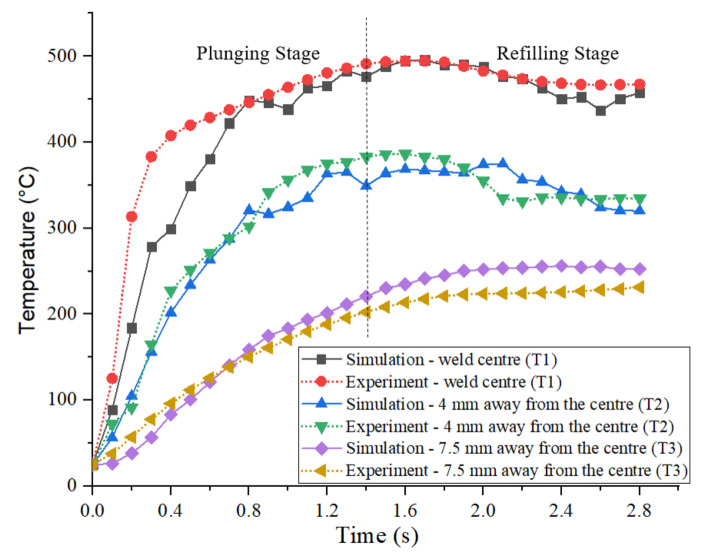
Comparison of experimental and numerical temperature results at different locations T1, T2, and T3 as indicated in Figure 3.

At first, the temperature field simulation results are compared to the experimental temperature field results at the weld centre, 4 mm and 7.5 mm outward from the weld centre, see Figure 6. A good agreement for the thermal cycles is obtained at these three points. The maximum temperature recorded at the weld centre in the experiment and simulation is around 495 °C. At the beginning of the plunging stage, a steep rise of temperature is observed up to 0.3 s of the process. Thereafter, a gradual increase of temperature is determined, which then gradually drops towards the end of the process in the weld zone. Although the temperatures drop with increase in distance from weld centre for the analysed points, the behavior of the temperature curve at 4 mm outward from the weld centre is still identical to that of the weld centre. At 7.5 mm outward from the weld centre, a steep increase, in the beginning, is no longer visible, i.e., the temperature gradually increased during the process time.

**Figure 7:**
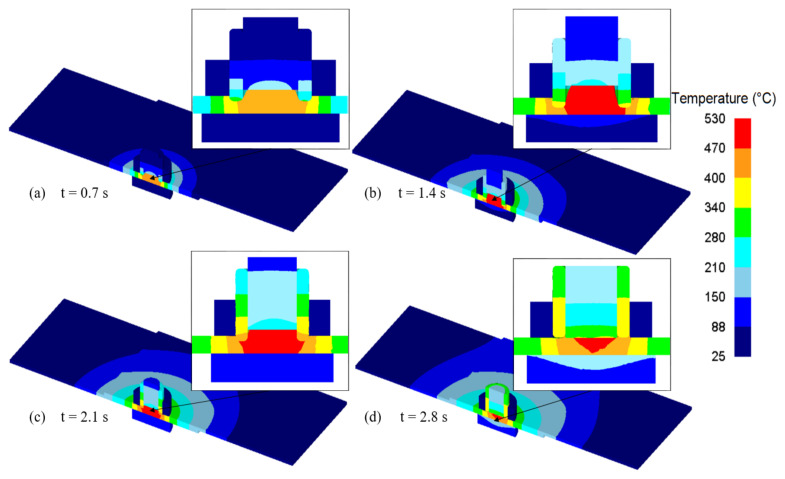
Temperature distribution during plunging (**a**,**b**) and refilling stages (**c**,**d**) of refill FSSW. (**a**) 0.7 s; (**b**) 1.4 s; (**c**) 2.1 s; (**d**) 2.8 s.

The temperature is strongly connected to the amount of generated heat, which again has impact on the stirring and softening of the material during the refill FSSW process. The spatial temperature distribution during the refill FSSW simulation is presented in Figure 7, which shows an axisymmetric distribution with respect to the weld centre. It can be seen that the maximum temperature during the refill FSSW process was reached inside the shoulder periphery, where the maximum temperature during the process is determined as 530 °C at a process time of 1.6 s. This is roughly 83.5% of the material melting temperature, which is a value typically reported for friction stir welding [33]. As can be seen from the temperature trajectories, there is a dip in peak temperatures as the span from the weld centre increases.

**Figure 8:**
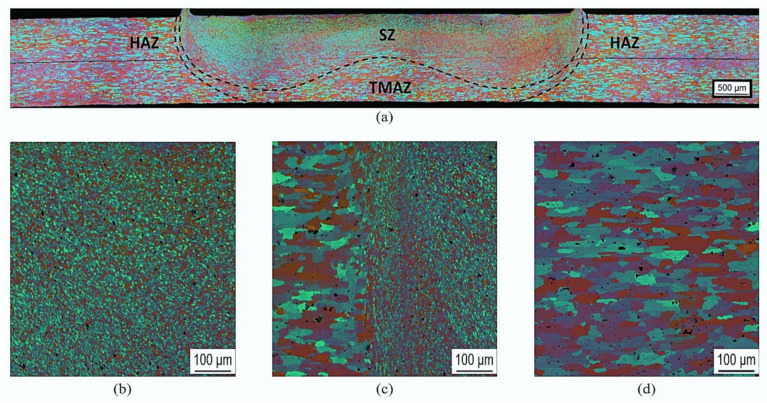
Microstructure of refill FSSW joint. (**a**) Cross-section of the weld; (**b**) stir zone (SZ); (**c**) thermo-mechanically affected zone (TMAZ) and SZ interface; (**d**) base material.

**Figure 9:**
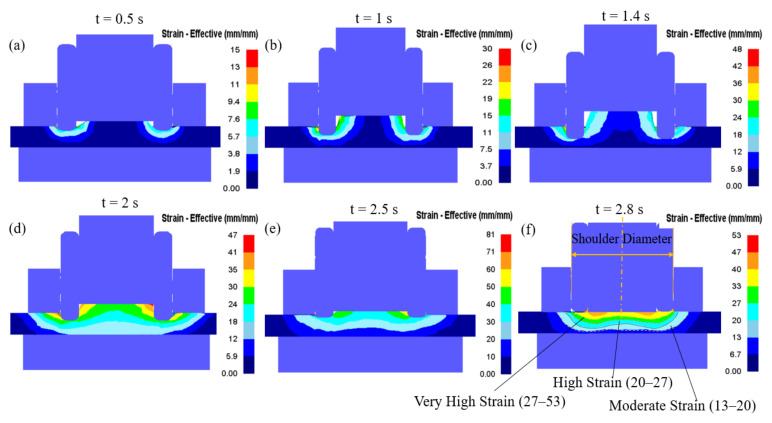
Strain (effective) distribution during the plunging (**a**–**c**) and refilling (**d**–**f**) stages. (**a**) 0.5 s; (**b**) 1 s; (**c**) 1.4 s; (**d**) 2 s; (**e**) 2.5 s; (**f**) 2.8 s.

In refill FSSW, as it is a thermo-mechanical process, temperature, strain, and strain rate plays a vital role in deciding the final microstructure, for instance, the grain size. Experimental micrographs after refill FSSW are presented in Figure 8. The refill FSSW weld appears completely filled and symmetric about the tool axis. The stir zone (SZ) can be distinguished with fine equiaxed grains, which are associated with dynamic recrystallization due to high plastic strains and temperatures in that region. Around the SZ, a narrow zone with highly deformed grains, which experiences moderate plastic strain is identified as a thermo-mechanically affected zone (TMAZ). As the strain in the TMAZ is moderate, dynamic recrystallization did not happen. The SZ and TMAZ boundary can be observed around the shoulder periphery. Although the characteristic zones can typically be identified from the micrograph, it is nearly impossible to obtain information about the strain and strain rate, which the material locally experienced, leading to the specific microstructure. However, this information is directly available from the simulation. The plastic strain distribution at different times during the process is presented in Figure 9. From the plastic strain contour, the area unaffected by tool stirring but affected by temperature, i.e., the heat-affected zone (HAZ), can be identified. The region outside the SZ with moderate plastic strain (13–20 mm/mm) is defined as TMAZ, where high plastic strains (20–53 mm/mm) are observed in the SZ. Although the plastic strain obtained from the simulation is consistent with the microstructure, there is an inconsistency of moderate plastic strain contour under the clamp region that is slightly wider than the TMAZ from the microstructure. Like the temperature, the plastic strain is symmetrical about the weld centre in refill FSSW. During the plunging stage, large plastic strain is mainly observed around the shoulder periphery. In the refilling stage, as the probe pushes the material in to weld, the strain also increased underneath the probe. Due to the shoulder-plunging variant, the region near the shoulder experiences the highest deformation during the process. The characteristics of the strain contour obtained from the simulation are comparable to the characteristic zones in the experimental micrographs. It can be seen from the micrographs that the grain size in the shoulder affected region is finer than the grain size in the weld centre, also reported in previous experimental studies [10,11,12,13], which is particularly related to the more severe plastic deformation.

**Figure 10:**
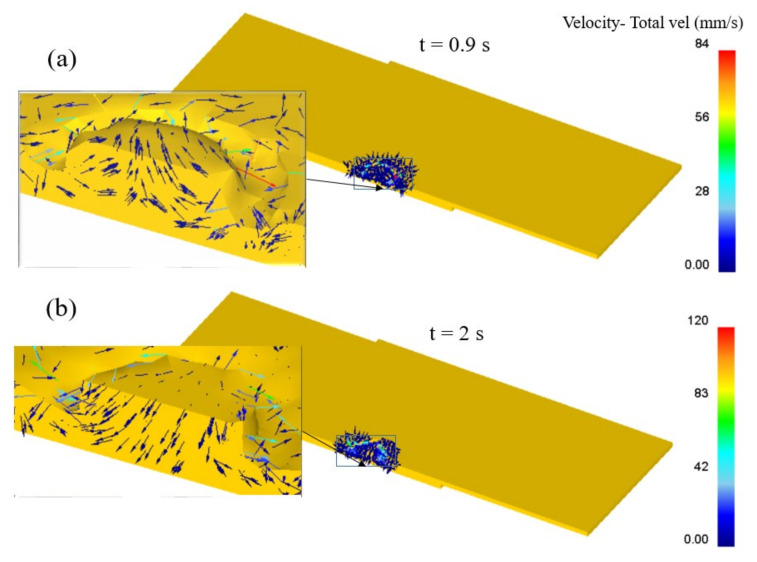
(**a**) Velocity vectors during plunging stage at 0.9 s; (**b**) Velocity vectors during refilling stage at 2 s.

**Figure 11:**
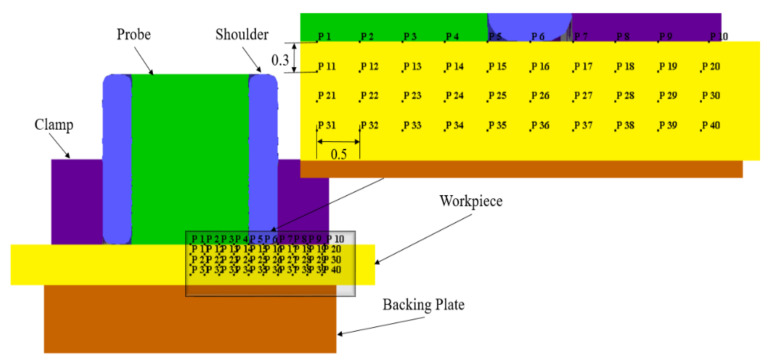
The initial position of points from the point tracking option that have been selected for studying the material flow.

Material flow is a vital aspect in determining the joint quality in the refill FSSW process [17,19]. Material velocity vectors during plunging and refilling stages are shown in Figure 10, giving a first idea of the material flow. However, to analyse the material flow in the weld area in detail, the behavior of selected points, as indicated in Figure 11, is investigated. As the process is symmetric, the points were initially located within a cross-section on one side of the cross section, starting from the weld centre up to a distance of 9.5 mm with a spacing of 0.5 mm between them. Additionally, the points were located at different heights along the sheet thickness with a vertical distance of 0.3 mm between them. P1–P10 represented points at the top surface of the workpiece, where P21–P30 are initially located between the top and bottom sheets interface. (Note again that top and bottom sheets are modelled as single sheet for simplicity).

**Figure 12:**
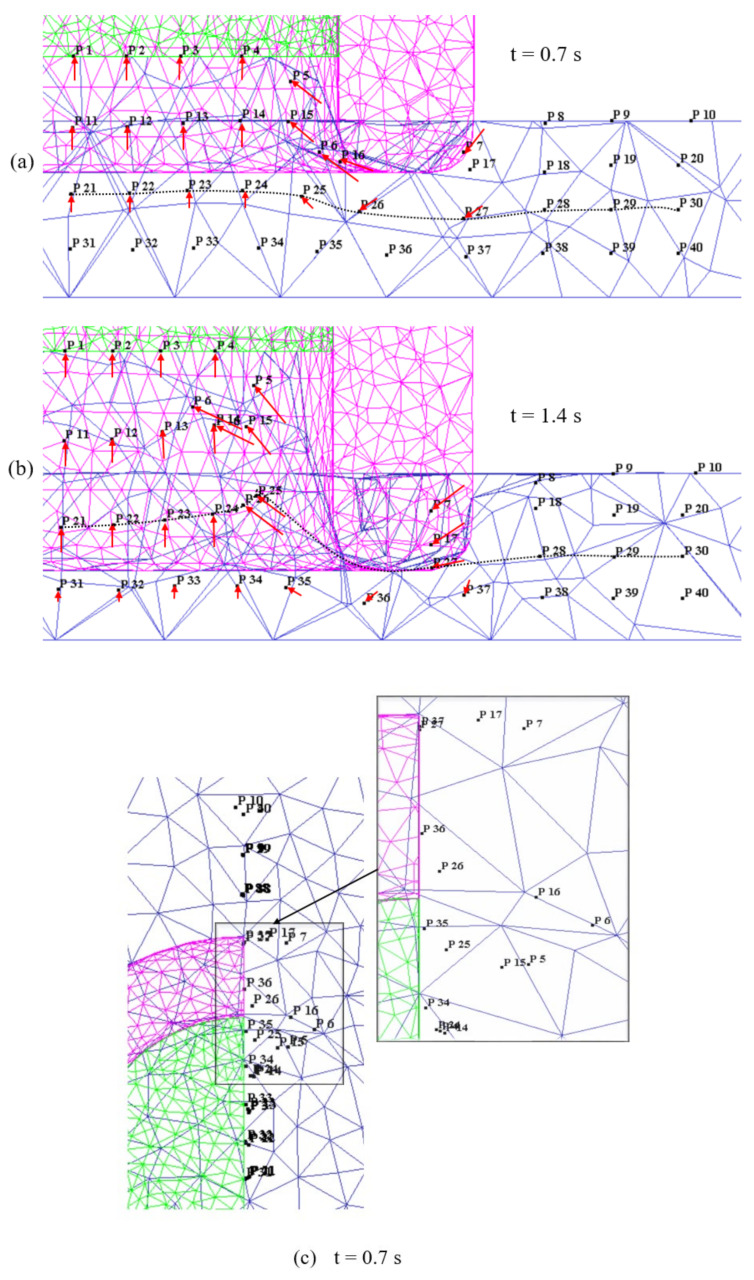

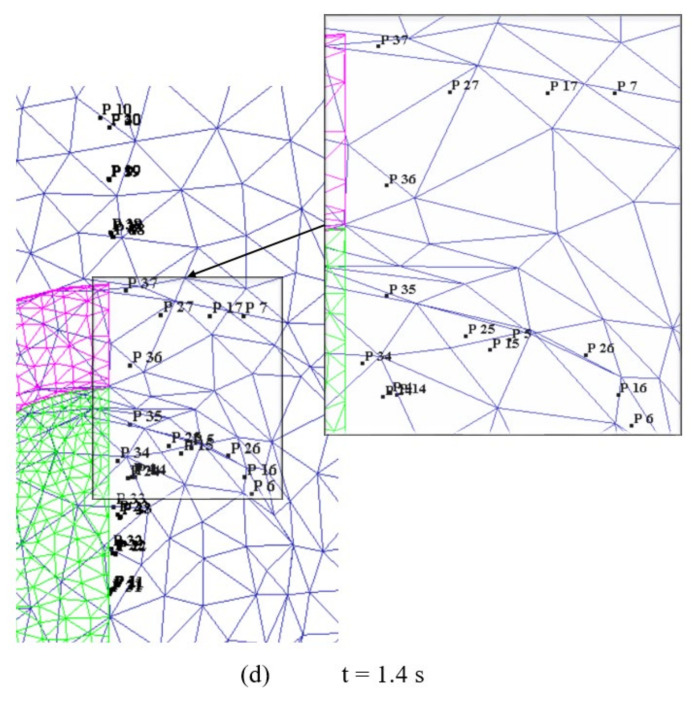
Point tracking positions representing material movement during plunging stage: (**a**) Front view −0.7 s; (**b**) Front view −1.4 s; (**c**) Top view −0.7 s; (**d**) Top view −1.4 s.

The material flow during the plunging stage is illustrated in Figure 12. During the initial phase of the plunging stage, the plasticized material is filled into the reservoir between the probe and shoulder. The material within the inner shoulder diameter moved upwards into the reservoir, see points P1–P4, P11–P14, and P21–P24. The material underneath the interior surface of the shoulder, i.e., points P5 and P15, was squeezed inwards into the reservoir and strongly sheared outwards, see the top view in Figure 12c. The material under the shoulder, i.e., points P6 and P16, and the material adjacent to the shoulder outer periphery, i.e., points P7 and P17, moved inwards and sheared outwards, see Figure 12a,c. At the halfway stage of plunging (t=0.7 s), i.e., when the shoulder has reached the depth of 0.35 mm, the material under the shoulder and adjacent to the shoulder outer periphery in the bottom sheet is pushed downwards, see P26 and P27 in Figure 12a. At this stage, the material in the bottom sheet shows minimal material flow. Figure 12b,d illustrates the material flow at the end of the plunge stage (t=1.4 s), i.e., the shoulder has reached the depth of 0.7 mm. The material within the inner shoulder diameter moved further up in the reservoir, see points P1–P4, P11–P14, and P21–P24. The material adjacent to the shoulder inner periphery, underneath the shoulder, and adjacent to the shoulder outer periphery is further squeezed towards into the reservoir, see points P5, P15, P25, P6, P16, P26, P7, P17, and P27. This material movement is influenced by the shoulder outer wall [34].

Overall during the plunging stage, the material inside the shoulder inner diameter shows an upwards motion towards the reservoir created by the moving probe. Material adjacent to the shoulder periphery is squeezed inwards and also sheared outwards, see Figure 12d. For the other areas of material outside the shoulder outer periphery the material movement is negligible, see points P8–P10, P19–P20, P29–P30, and P38–P40. Most of the material of the bottom sheet, i.e., points P31–P40, has no significant movement except for the material around the shoulder periphery, see points P35, P36, and P37. The shape of the interface and the observed material flow during plunging and the material flow directions are consistent with experimental results reported in the literature [17,34].

**Figure 13:**
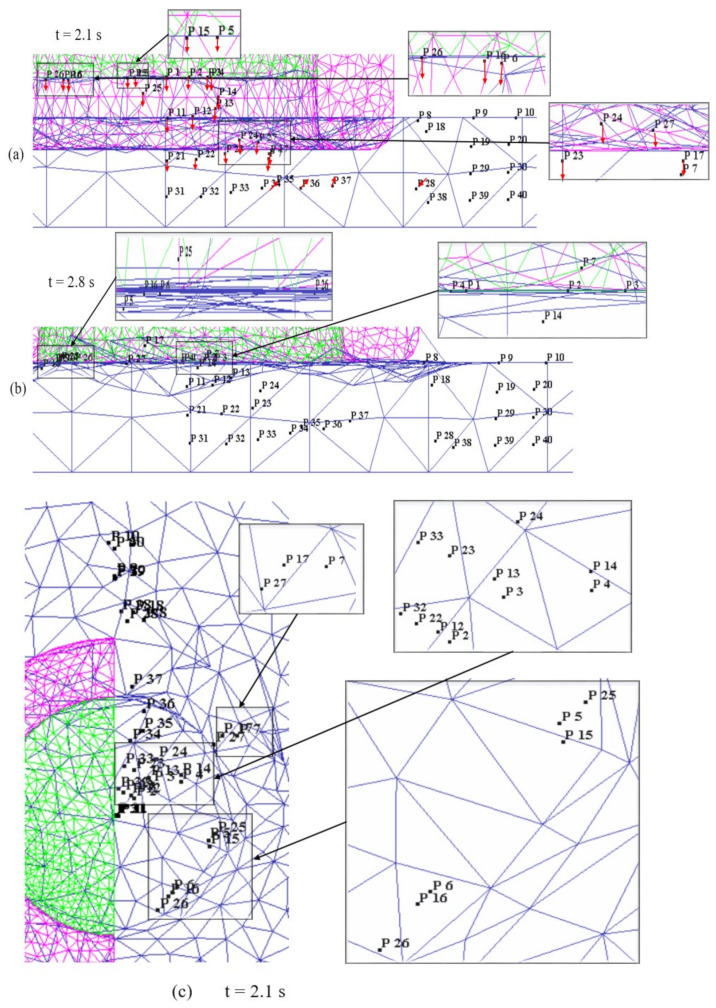

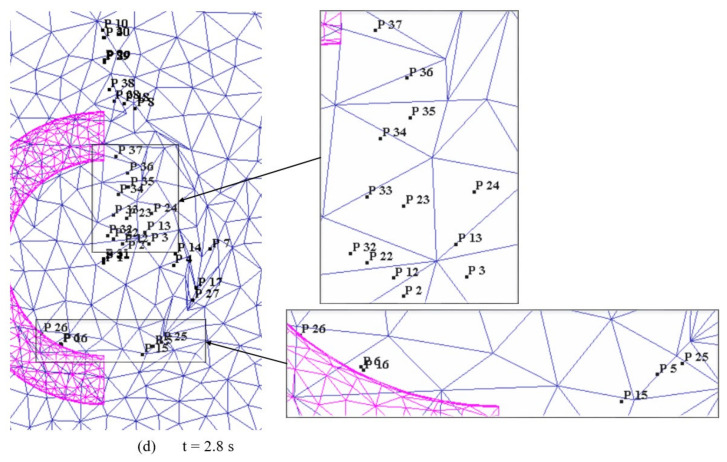
Point tracking positions representing material movement during refilling stage: (**a**) Front view −2.1 s; (**b**) Front view −2.8 s; (**c**) Top view −2.1 s; (**d**) Top view −2.8 s.

The material flow during the refilling stage is illustrated in Figure 13. The material in the reservoir is refilled into the weld region by the rotating probe. The material, which is accumulated in the reservoir during the plunging stage, is pushed downwards by the probe, see Figure 13a,b. The material from the bottom sheet, around the shoulder periphery moved upwards as the shoulder is retracted, see points P35, P36, and P37 in Figure 13a,b. The material outside the shoulder outer periphery moved inwards slightly as the shoulder moved upwards, see point P28. The movement of material around the shoulder periphery is indicating intermixing of material between the top and bottom sheets, see points P5–P7, P15–P17, and P25–P27. The material inside the reservoir is sheared outwards slightly due to action of rotating probe, see Figure 13c,d. The material around the shoulder’s inner periphery and under the shoulder strongly sheared outwards due to the rotation of the shoulder, see points P5–P7, P15–P17, and P25–P27 in Figure 13c,d. At the end of the refilling stage, a flat weld is produced as seen in Figure 13b. Overall during the refilling stage, the material in the reservoir is pushed back into the weld and the material from the bottom sheet moved upwards due to shoulder retraction and pushing of material from the probe. The material outside the shoulder outer periphery remained unaffected throughout the process, see points P8–P10, P18–P20, P29–P30, and P38–P40. The material movement mostly occurred in the SZ within the shoulder periphery region. The material movement outside the shoulder periphery occurred in a quite narrow zone, i.e., TMAZ, during the plunging and refilling stages, see points P7, P17, P27–P28, and P37. For the material outside the shoulder periphery, i.e., located 4 mm and 4.5 mm away from the weld centre, material flow was negligible.

**Figure 14:**
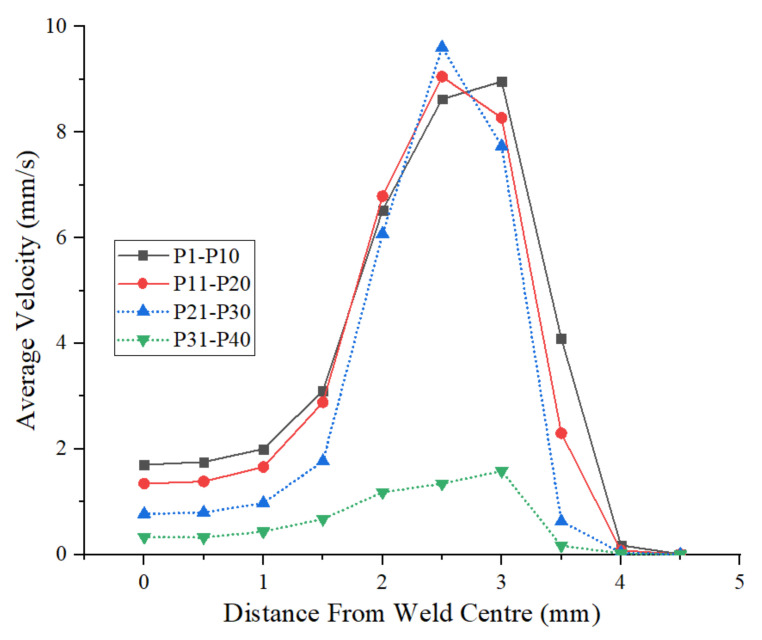
Average velocities of the points (P1–P40), located for material flow study.

To understand the local material flow behavior further, the time-averaged velocities of the analysed points for material flow i.e., points P1–P40 during the refill FSSW are shown in Figure 14. The results illustrate that the material near the weld centre has lower average velocities and the maximum velocities are observed for the material adjacent to the outer radius of the shoulder and under the shoulder, consistent with previous studies [19]. The maximum average velocities are obtained at a distance of 2.5 mm and 3 mm outward from the weld centre, i.e., P6, P7, P16, P17, P26 and P27 under the shoulder and shoulders outer periphery, where the point at the interface, P26, shows the maximum average velocity (9.6 mm/s). The average velocity of the material within the bottom sheet is low compared to the material velocities within the top sheet. The maximum average velocity is found to be in the region adjacent to the outer periphery of the shoulder, P36, due to the displacement of the bottom sheet material towards the shoulder cavity while the shoulder is retracting during the refilling stage. From the material flow and material velocity results, it can be seen that there is no significant movement of the material after a distance of 3.5 mm outward from the weld centre.

**Figure 15:**
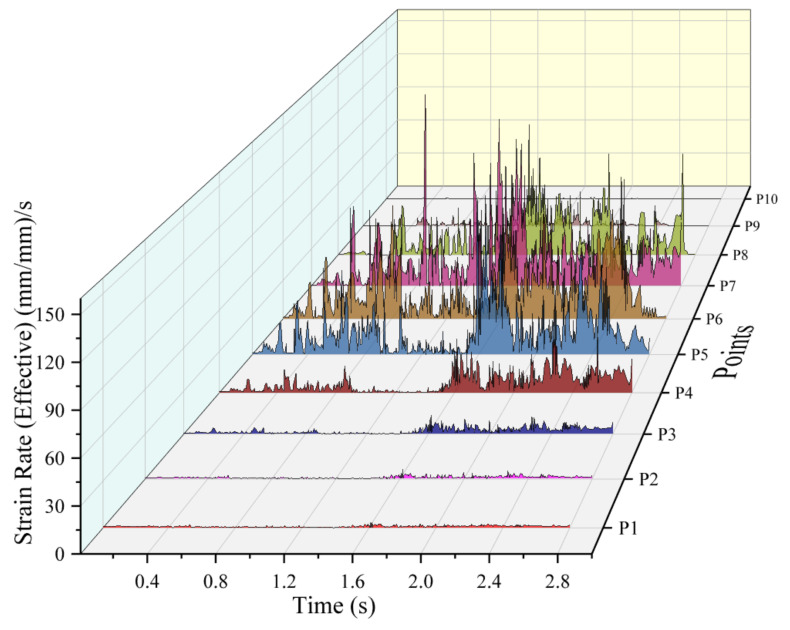
Strain rate at the point locations (P1–P10), located on top of the workpiece, see Figure 11.

Although the material velocities are of high interest to understand the observed flow behavior, the driving factor for the material response, for instance in terms of microstructure evolution, is the strain itself. To illustrate this in more detail, the strain rate during the process at the point locations P1–P10 is shown in Figure 15. The strain rate is predominantly observed around the shoulder periphery. The material adjacent to the shoulder inner diameter, P5, material under the shoulder periphery, P6, and the material adjacent to the shoulder outer diameter, P7, experience high strain rates. The strain rate trends can be compared to the velocity graph discussed earlier. The deformation of the material largely occurs around the shoulder periphery. For the material inside the shoulder inner diameter the intensity of strain rate is minimum, see points P1–P4. As the distance from the shoulder periphery increased, the intensity of strain rate decreased, see points P1–P4 and P9–P10. The strain rate in the material outside the shoulder outer diameter can be seen up to a distance of 3.5 mm from the tool central axis. There is negligible strain rate for the material at a distance of 4.5 mm outward from the weld centre, i.e., point P10. This is an indication that there is no mechanical deformation in that region.

**Figure 16:**
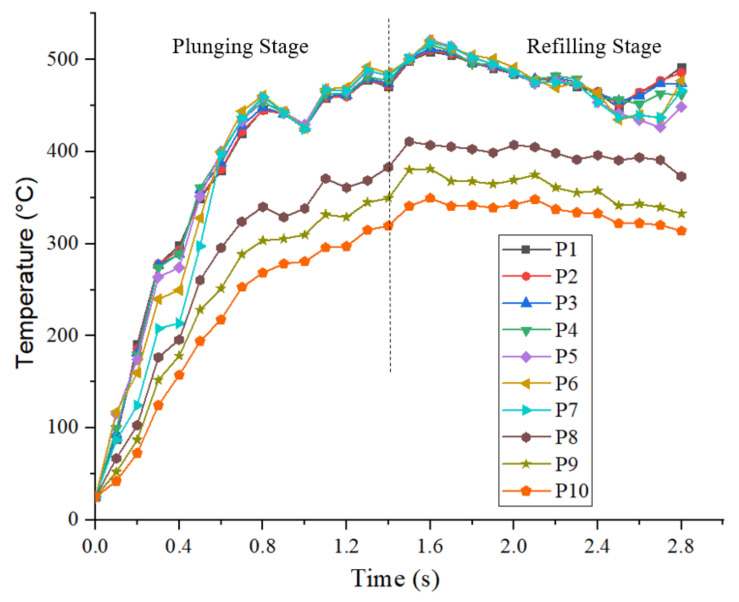
Temperatures at the point locations (P1–P10), located on top of the workpiece, see Figure 11.

As the material flow study showed, the joint consists mainly of material within a radius of 3.5 mm from the weld centre (for a shoulder diameter of 6 mm). Since in Figure 6, next to the temperature at the weld centre, only the temperature with distances more than 4 mm outward from the weld centre were analysed, the temperatures for the point locations P1–P10, marked on top of the workpiece, are analysed in Figure 16 in detail. All material within the stir zone i.e., points P1–P7, shows nearly the same thermal cycle, congruous with the results in Figure 7. The peak temperature in the SZ is reached at 1.6 s of the process. Gradual increase of temperatures is observed up to 1.6 s of the process, and from thereon temperatures dropped gradually. For the region outside the SZ, i.e., P8–P10, lower temperatures are observed. From the temperature profiles, it is seen that the temperatures in the stir zone crossed the solidus temperature (475 °C) [35] and reached a peak of 521 °C. The hardening precipitates in the base alloy such as η′ (MgZn_2_), dissolves thereby soften the material in the stir zone more than the other zones [36]. Due to this softening, the shearing of the material in the stir zone is enhanced. Also, these high temperatures boost the diffusion bonding between the sheets [35].

**Figure 17:**
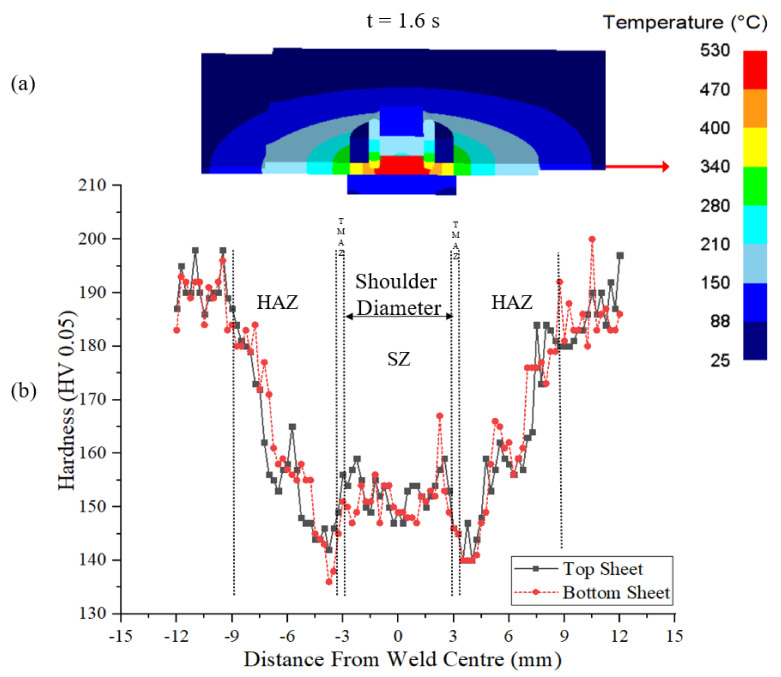
Hardness profile of the top and bottom sheets of the weld (**b**) after welding compared to temperature distribution at t = 1.6 s (**a**).

Finally, Vickers hardness tests were performed horizontally within the cross-section at the middle lines of the top and bottom sheet. The results are shown in Figure 17b. The inherent isomorphous precipitates condition after the welding becomes a dominant factor of the microstructure to control the resultant hardness properties. This is because of the effect of intense plasticization on the extent of precipitates dissolution during stirring [37,38]. The measured hardness profile is symmetric about the weld centre and shows a typical ‘W’ shape, which corresponds to obtained hardness profiles of AA7075 refill welds in the previous studies [8,10,39]. The hardness values of the base material are around 190$HV_{0.05}$. Hardness reduction is present in the HAZ. The drop of the hardness of HAZ can be connected to the coarsening of fine strengthening precipitates in the matrix due to the heat generated during joining. The temperature distribution at 1.6 s of the process, i.e., the instance of peak temperature in the SZ in the process is shown in Figure 17a. HAZ experiences only a thermal cycle and the temperature from the simulation in this zone is less than 410 °C, see Figure 16. At this temperature, the dissolution of hardening precipitates is incomplete. The hardness dropped to the merest of values at the HAZ and TMAZ interface and then increased from TMAZ to SZ. A similar trend was observed in earlier studies [8,10,39]. The range of TMAZ is narrow on either side of the shoulder’s outer diameter. As discussed earlier TMAZ experiences moderate plastic strain and strain rates, see Figure 9 and Figure 15. The peak temperature observed in the TMAZ is 470 °C, which is marginally below the solidus temperature of the material. The hardness value slightly rises in the SZ from the TMAZ. The SZ experiences higher temperatures, above the solidus but lesser than the melting temperature of the material, see Figure 16. The maximum temperature in the SZ is 530 °C and the SZ involves high plastic strains. The dissolution of hardening precipitates of *η*′ (MgZn_2_) occurs to a larger extent considering the range of temperatures in the SZ [39,40]. The stir zone has the greatest capability for reprecipitation and hardness improvement during natural ageing [41].

## 4. Conclusions

A 3-D numerical model is presented for the refill FSSW process using an updated Lagrangian formulation by employing DEFORM-3D. The model is able to deal with severe element distortion via extensive remeshing. The numerically obtained temperature distribution is in good agreement with the experimental results of thermocouples. The temperature distribution is symmetric about the weld centre and the maximum temperature during the process is slightly above the reported solidus temperature and reaches 83.5% of the melting temperature of the material. The distribution of plastic strain is symmetric about the weld centre as well, where the maximum plastic strains are observed in the region around the shoulder. The microstructure evolution and shape of the stir zone from the experiment is correlated to the thermal distribution and the plastic strain distribution obtained from the numerical model. Using point tracking, the material flow is investigated, where significant movement of the plasticized material is identified in the SZ. Intermixing of the material between the top and bottom sheets is visible around the shoulder periphery. The maximum movement of material is observed adjacent to the shoulder outer periphery. The material flow is negligible after a distance of 4 mm outward from the weld centre. The maximum strain rates were observed for the material around the shoulder. The SZ, TMAZ, and HAZ can be distinguished using the simulation results. The hardness profile from the experiment appeared in a ‘W’ shape and the hardness reduction in the zones was correlated to the temperatures from the simulation. It is evident from the results that the presented 3D numerical model is capable of predicting the temperature, material flow, strain, and strain rates well during the refill FSSW process, allowing an in-depth investigation of experimental observation.

## Figures and Tables

**Table 1:** Temperature-dependent friction coefficient between steel and aluminium [32].

**Temperature (°C)**	20	160	200	400	500	580
**Coefficient of Friction (µ)**	0.35	0.3	0.26	0.08	0.03	0.01
